# Life; an Essay

**Published:** 1890-02

**Authors:** W. A. Morris

**Affiliations:** President Austin District Medical Society


DANIEL'S

### TEXAS MEDICAL JOURNAL.

A Representative Organ of the Medical Profession, and an Exponent of Rational Medicine; devoted to the Organization, Advancement and Elevation of the Profession in Texas.

PUBLISHED MONTHLY.—SUBSCRIPTION $2.00 A YEAR.

Vol. V. AUSTIN, FEBRUARY, 1890. No. 8.

### Original Articles.

☞CONTRIBUTED EXCLUSIVELY TO THIS JOURNAL.☜

*The Articles in this Department are accepted and published with the understanding that we are not responsible for, nor do we indorse the views and opinions of the writers by so doing.*

### LIFE.

[Retiring Address of W. A. Morris, M. D., President Austin District Medical Society, delivered at the Ninth Quarterly Meeting, at Austin, Dec. 20, 1889.]

IT HAS been written on the pages of the most ancient and grandest of all books, that God created man of the dust o the earth and breathed into his nostrils the breath of life, and man became a living soul. On the pages of the same book the immutable decree has also been written: Dust thou art and unto dust thou shalt return. Ever since then man has been seeking an elixir that would annul the decree and cause him to bloom in perpetual youth, but in this age of light and progress we look no longer for that elixir; but to us the representatives of the present the glorious privilege has been assigned to seek the best means of curing the ills of man, and to secure to him the greatest length of days which it is possible for him to attain.

It becomes us, then, to inquire, what is that living principle which animates our mortal bodies; what is life?

"God created man of the dust of the earth and breathed into his nostrils the breath of life, and man became a living soul,"

Life is a mystery that has never been solved; a problem that the finite mind cannot comprehend. Many definitions have been given, all equally unsatisfactory.

An able scientist has expressed it thus: "Life itself is only physical and chemical force transformed, or physical and chemical force transformed into vital force, and vice versa."

This definition, like all others, fails to throw any light upon the subject since it identifies life's phenomena with life itself.

We can only witness the manifestations of life and study its laws. All that we can behold or contemplate in the physical universe presents itself under two forms, one living, and the other dead matter.

The diffusion of life is almost universal. It has been found wherever man has been; it exists in the polar regions, imbedded in snow and ice that we know not how long have resisted the rays of the sun; it is found in the depths of the ocean, where eternal night reigns, and the light of the sun never penetrates. It exists in every form, and adapts itself to every condition.

Small organisms have built up large islands in mid-ocean. This infusorial life has largely contributed to the formation of the crust of the earth, and this little microcosm of ours is a storehouse for their dwelling place. It is with the life of man, however, that we have to deal. Although we cannot lift the curtain and unfold the mystery that it hides from our view, yet it is strictly within the domain and bounden duty of every scientific physician, to study well the laws that govern the human organism in both health and disease.

"Know then thyself.
Presume not God to scan,
The proper study of mankind is man."

Let us take a survey of this globe of ours with all its wonderful phenomena, and then lift our eyes to our great solar system, and to other suns and systems beyond.

The minds of earth's greatest philosophers are lost in amazement while beholding their beauty, grandeur and glory.

It is said of Newton that when he first used an improved telescope that gave him a greater reach of vision, and brought new stars and new system to view, he was struck with such astonishment and awe, that it was some time before he could resume his studies.

Although the architecture of the universe is grand beyond our comprehension, man is far a grander work in the scale of creation.

The one is inorganic matter, the organic and organized, with mind associated in some occult manner unknown to us. A most profoundly complex being. In him, then, you have a subject worthy of the highest faculties of the mind, and to you is assigned the task of studying that wonderfnl organism, the masterwork of creative genius.

The watchmaker must be familiar with every part of its machinery, or he will not be qualified to repair it when out of order. The engineer must understand the science of engineering. The pilot must know all the dangers in his pathway. or he cannot be entrusted to conduct his vessel safely to its destined port.

The human system is, however, unlike any machinery of man's make. It has been likened by some to a steam engine governed by correllated forces which are all from without. There is nothing inherent. The machine is only dead matter operated upon by mind, and is wholly at the will of the operator.

The analogy soon ceases, and then diverges to meet no more. It has also been likened to a chemical laboratory, governed by chemical laws. This is a great mistake. Life or vital force controls these chemical affinities, and makes them subservient to the well being of the individual.

The amoeba represents the lowest form of animal life, a living cell. The growth of all organized bodies is the result of cell life, and each individual cell is the product or parent of another living cell.

All life that is upon the globe has been perpetuated from the beginning by this unbroken chain.

In the animal kingdom, the growth of every cell that goes to build up the organism is dependent upon digestion and assimilation.

When prepared to enter the circulation, it is taken up and carried by the great blood current to the different tissues of the body, and with unerring certainty to its place of destination, whether to bone, muscle, cartilage, skin, brain or nerve. As soon as the cells have subserved their purpose, their oxydation, disintegration and death take place, and as dead effete matter, are carried off through the different channels of the body.

Repair and waste, life and death, are constantly going on in the living organism. Life forces control other forces, and when death takes place, by disease or age, chemical affinities come then into play and assert their supremacy, and all the different elements of the once living organism fly away to unite with more congenial attractions.

The chemical theory is wholly inadequate to account for cell life, its structure, composition, or mode of action in building up living tissue.

The spermatozoon, as if guided by inherent wisdom, makes its way to the ovum, and finding its habitat, by coalescing, it forms a new being.

The salivary glands secrete ptyaline, a ferment precisely adapted for the preparation of food for the stomach. The gastric juice is ready prepared to act upon the food and convert it into peptones. The pancreatic juice and the bile are ready to complete the digestion process, and prepare the nutritive properties of the food for absorption and assimilation.

None of these can be accounted for by any known chemical law, nor can it explain why the gastric juice will not attack the living stomach, but will often invade its coats soon after death.

A contemplation of these subjects brings forcibly to our minds, although we have learned much in the past, how meagre our knowledge is yet. Nearly fifty years ago it was said by a profound thinker, "Large as is the area of our knowledge, the area of our ignorance is much larger; that the unknown incomparably transcends the known in all the departments of true science."

The discoveries since that time have been most wonderful in all the departments of scientific research, fully verifying the truth of the propositions.

Pardon the digression while I take a cursory view of what time has done since I entered upon the stage of action. It is oft times pleasant to meditate upon the past and live over again the scenes of early life and manhood. In youth, our thoughts are intent upon the days to come; but as we grow old, we live again in those that are past.

In the beginning of the last war with Great Britain, in 1812, our population was about seven millions. Hostilities continued for nearly three years, and ended with the battle of New Orleans, in 1815. The great valley of the Mississippi was then almost a wilderness, and to and beyond the Rocky Mountains a savage wild. We now have a population of over sixty millions, and farms and cities dot our country from the Atlantic to the Pacific coast, and from the Lakes of the North to the Rio Grande. Our commerce is on every ocean, and our flag commands respect from every nation.

In the year in which I was born (1812) the first steamboat was launched upon the Mississippi river. Steam is now the great motive power of the world. Ships propelled by this mighty agent are floating upon the waters of every nation.

In 1831, the first railroad was built in the United States which was run by steam—a line of only fifteen miles—the Baltimore and Ohio. Now there are enough miles of railroad in the United States to belt the earth six times.

Printing was a slow and tedious process, being done by hand. The Hoe printing press was invented since I began my professional career, which turned off ten thousand sheets an hour, which was then considered a most remarkable achievement. Now, with new methods, seventy-five thousand sheets can be printed in one hour—over a million a day.

Franklin, in the last century, stole the lightnings of heaven from the clouds by means of a kite; Morse put it in harness to do the work of man, and on the 24th day of May, 1844, a message was sent from Washington to Baltimore. More recently a pathway was made in the depths of the ocean, and it is now controlled by the will of man. We bid it start on a message round the world, and it beats the sun in his course.

By the mighty genius of an Edison we are lighting up our firesides and cities, and propelling our engiues, and can converse with our friends hundreds of miles distant,—the tones and modulations of the voice understood.

More wonderful still, the speeches of our friends, or distinguished men, can be stored away and reproduced at will by an inanimate machine, with all the intonations of the voice of the one who delivered it.

It has also become a very important agent in the hands of scientific physicians in the cure of disease.

Not many years ago a little child touched a small button. Soon there was a sound as of thunder and the whole city of New York was in commotion. At that moment an immense rock that had long obstructed the harbor, was shivered to atoms, and the obstruction of Hell Gate gave way. The touching of that button by a small child controlled the forces that did it—dynamite and electricity.

In the year of 1800 gas was unknown. The discovery of the spectroscope and spectrum analysis is of recent date which has enlarged our field of observation and disclosed to us wonders that we had not before conceived of.

The discovery of the manufacture of ice, which has added so much to our comfort in health and disease, is within the recollection of nearly all who are present.

Archæological explorations have carried us back into the night of time, and the dead past has been made to speak and tell the story of vanished races of pre-historic ages.

Immense cities have been exhumed. The site of Ninevah was discovered by a French savant as late as 1846. In 1848-9 excavations were made by Layard and others which disclosed to us the magnificence of her departed glory and grandeur. In her deep recesses inscriptions were found giving to the world a history of her kings, her battles and her victories, confirming Biblical history relating to her times.

Troy, which existed only in song, was discovered by Schliemann, buried deep under the ground; her richest treasures have been made known to the world after a lapse of three thousand years.

Champollion, in 1822, got a glimse of the key which enabled him ultimately to decipher the Egyptian hieroglyphics. In 1828 he was sent by the French government on an archæological exploration, in company with an Italian, deputed by the Italian government. The exploration was crowned with success and gave to the world the earliest records that were ever given to man, the great value of which can not be over-estimated. In 1831 he died, full of honor and clothed with a nation's gratitude.

The physiology of digestion was very little understood until Liebig shed light upon the subject. Since that time our knowledge of organic and physiological chemistry has enabled us to trace the changes that take place from the time the food is placed in the mouth and while passing through the different organs, till the process of digestion is completed; and it has furnished us with many valuable agents by which we can aid the digestive process, and even digest food outside the body and prepare it already for absorption. The discovery of ptomaines is of recent date.

Pathological anatomy was then comparatively in its infancy. Now we can, with much certainty, read from the symptoms the pathological changes that are taking place in the different organs of the body—a knowledge of which is necessary to a correct diagnosis, and a correct diagnosis is essential to a correct prognosis and treatment.

Gynecology, as a separate branch of study, was not known, and the most of the diseases of the uterine system were treated symptomatically.

Although the speculum is an ancient instrument, it was scarcely ever used in practice. Of late years it has been of invaluable use in enlarging our knowledge of the diseases of the womb.

The knowledge which we have gained in that department and the scientific skill with which important operations are performed successfully, has challenged the admiration of the world.

Now thousands of lives are saved that then would have been consigned to suffering and death. Some of the most important operations, such as for vesico-vaginal fistula, ovariotomy, Porro's operation, etc.

Chemical science has unlocked the great storehouse of nature, and revealed to us her most wonderful and secret treasures, and has given us many of our most valuable remedies, such as the iodides and bromides, chloral, chloroform, cocaine, nitrite of amyl, nitro-glycerine, and nearly all of the alkaloids, and that valuable class of remedies we call antipyretics and hypnotics, of which latter sulfonal promises to be a valuable representative.

In surgery the advance has been grand beyond conception, especially in that of the abdomen and brain. Fifty years ago it would have been madness to have attempted an exploration of the brain or abdominal cavity. But recently almost every organ of the body has been made to pay tribute to science, except the heart and medulla oblongata.

Our knowledge has also been very much enlarged in therapeutics. Many new remedies have been made known to us of great value. Some have already been named.

Then the therapeutic properties of ergot were not understood. Jaborandi is of recent discovery and an agent of great value. But with all that there is yet a very wide field to be explored in this department, and much to learn in the modus operandi of remedies in the cure of disease.

In 1824 Dr. Bright first called the attention of the profession to a fatal form of disease of the kidney which was previously unknown, now called Bright's disease, in honor of his name.

Many other diseases have since been made known to us. Asiatic cholera first made its appearance on our continent in 1832. The invasion of no hostile army in any country ever produced more consternation. Diphtheria and malarial hematuria were not recognized then.

The etiology of disease is much better understood by the aid of the microscope,—wonderful revelations have been made, especially in bacteriology, which have opened to us a vast field for scientific investigation and placed us far in advance of former years. The antiseptic treatment of wounds, and in surgical practice and sick-rooms, the value of which no one will question, is one of the things which has taken up its abode with us and will remain.

[CONCLUDED IN NEXT ISSUE.]

